# LAMP3 plays an oncogenic role in osteosarcoma cells partially by inhibiting TP53

**DOI:** 10.1186/s11658-018-0099-8

**Published:** 2018-07-11

**Authors:** Shaoxian Liu, Junyi Yue, Wei Du, Jian Han, Weidong Zhang

**Affiliations:** 1 Department of Bone Traumatology, Yantaishan Hospital, Yantai, 264000 Shandong Province People’s Republic of China; 2 Department of Spinal Research, Yantaishan Hospital, Yantai, 264000 Shandong Province People’s Republic of China; 3 Department of Bone Tumor, Yantaishan Hospital, Yantai, 264000, Shandong Province People’s Republic of China; 4 Department of Bone Traumatology, Yantai Hospital of Traditional Chinese Medicine, Yantai, 264000 Shandong Province People’s Republic of China

**Keywords:** Osteosarcoma, LAMP3, TP53

## Abstract

**Background:**

Osteosarcoma (OS) is a common malignant tumor that predominantly occurs in adolescents. Its most common metastasis is to the lungs. As shown in our earlier study, lysosome-associated membrane glycoprotein 3 (LAMP3) is highly upregulated in metastatic OS. However, its role in the regulation of OS cell viability and apoptosis remains unknown.

**Methods:**

We knocked down and overexpressed *LAMP3* in OS cells and assessed the cell viability and apoptosis. Then, we investigated the expression of apoptosis-associated genes to identify the downstream gene(s) of *LAMP3*.

**Results:**

Knockdown of *LAMP3* significantly inhibited OS cell viability and promoted apoptosis. *TP53*, which is involved in the apoptosis pathway, was found to be highly upregulated after knockdown of *LAMP3*. Overexpression of *LAMP3* significantly increased cell viability and abrogated apoptosis. Importantly, subsequent knockdown of *TP53* partially suppressed the increased OS cell apoptosis induced by the inhibition of *LAMP3*, suggesting that *TP53* is a key functional downstream gene of *LAMP3*.

**Conclusions:**

Our findings suggest that *LAMP3* promotes OS cell viability and survival by regulating *TP53* expression.

**Electronic supplementary material:**

The online version of this article (10.1186/s11658-018-0099-8) contains supplementary material, which is available to authorized users.

## Introduction

Originating from mesenchymal cells, osteosarcoma (OS) is a very common tumor occurring in the long bones. It presents with spindle cells and aberrant osteoid formation [1]. Exposure to radiation is a known cause, and it coexists with other disorders, such as Li-Fraumeni disease and hereditary retinoblastoma [2]. The first peak of OS incidence is in 10- to 16-year old adolescents, while the second peak occurs in people over 50 years of age [2, 3]. The overall world incidence is 1 to 3 cases per million people per year [4].

The survival rate of OS patients has been improved to a certain degree with conventional therapies, including preoperative chemotherapy, surgical resection and postoperative chemotherapy. This improvement is also thanks to identifications of potential prognostic factors and advances in clinical therapy and OS research. However, patients with OS recurrence or an advanced stage of the disease always show poor responses to these conventional chemotherapies, with most relapsing [4, 5].

In addition, OS often metastasizes via the hematogenous route to other organs, such as the lungs (95%), kidneys (12%) and other bones (50%) [6, 7]. Advances in clinical technology have significantly increased the survival rates of patients with localized OS (by more than 50%). However, most patients had less than a 20% survival chance when metastases occurred [2, 8]. For example, the 5-year survival rate for OS patients with metastasis to the lungs was just 11% when they were treated with high-dose methotrexate, doxorubicin, bleomycin, cyclophosphamide and dactinomycin, together with surgery. This only increased to 11.8% when combined with treatment with carboplatin and ifosfamide [9]. The mechanism of OS metastasis clearly needs more study.

Gene therapy and targeted treatment have gained the attention of the medical community in recent years due to their potential to elucidate disease mechanisms and affect change at the molecular level. Identification of specific genes is critical for their success.

Some molecular targets involved in OS metastasis have been found. *APEX1* and *HER2/neu* (*ErbB2*) were found to correlate well with recurrence and metastasis in OS patients [10]. *NFIB* was confirmed to be associated with metastasis in OS patients and the TP53 pathway was not only determined to be involved in primary OS development but is also a key factor for metastasis [11]. Several biopathways, including the HIF-1α & AP-1 and ERK & PI3K/AKT pathways, were also confirmed to be highly relevant for OS metastasis [11].

*LAMP3* is a member of the lysosome-associated membrane glycoprotein (LAMP) family. It codes a 416-amino acid protein [12]. It is primarily reported in lung tissues, and it is found to be overexpressed in several primary cancers, such as breast, lung and liver cancer [13]. Furthermore, *LAMP3* is correlated to the hypoxia regulation progress, which makes it a good biomarker for breast cancer [14], and epithelial LAMP3 expression is reported to be a prognostic biomarker for esophageal squamous cell carcinoma [15]. Importantly, *LAMP3* has been reported to be associated with many tumor metastases, such as cervical cancer and osteosarcoma [12, 16].

In our previous study, *LAMP3* was found to be one of the top upregulated genes in OS lung metastasis tissue compared to conventional OS tissue [16]. However, its role in the regulation of OS cell viability and apoptosis is still unclear.

*TP53* is widely accepted as one of the most important tumor suppressor genes. It acts as a central regulator of multiple biological processes, including cell proliferation and apoptosis [10, 17, 18]. Notably, *TP53* was proved to be an effective prognostic biomarker for OS patients [19]. Whether *TP53* is a crucial regulator of the *LAMP3* pathway in human OS remains to be investigated. Comprehensive understanding of its functional network will significantly benefit clinical treatment.

In this study, we investigated the impact of *LAMP3* on OS cell viability and apoptosis, and then identified the functional downstream gene of *LAMP3*. Our findings revealed that *LAMP3* increased OS cell viability and survival through the regulation of *TP53* expression.

## Materials and methods

### Cell culture and RNA interference

U2OS and OS-732 cell lines were respectively obtained from ATCC (American Type Culture Collection) and the Chinese Academy of Sciences. Cells were cultured in Dulbecco’s modified Eagle’s medium (DMEM) supplemented with 10% fetal bovine serum (FBS), 2 mM glutamine, 100 μg/ml streptomycin and 100 U/ml penicillin at 37 °C in a humidified atmosphere containing 5% CO_2_. To knockdown *LAMP3* and *TP53*, commercially validated si*LAMP3* and/or si*TP53* were synthesized by GenePharma and transfected to cells using Lipofectamine RNAi MAX (Life Technologies) according to the manufacturer’s instructions. Overexpression of *LAMP3* was accomplished through transfection to cells with expression plasmids from GeneCopoeia company (cat. no. EX-A6482-M02) using the Lipofectamine 3000 system (Thermo Fisher Scientific) according to the manufacturer’s protocol. Two days later, the medium was replaced with fresh full culture medium. After transfection for about 72 h, the cells were collected for western blot or quantitative RT-PCR.

### MTT assay

OS-732 and U2OS cells were seeded in 96-well cell culture plates with 2500 cells/well, and cultured for 24 h. Then, the cells were incubated with *LAMP3*/*TP53* siRNA for 48 h. After that, the cells were treated with 100 μl 5 g/l MTT for 4 h. 100 μl DMSO was added to each well 15 min before analysis. OD_570 nm_ was measured. Each assay was repeated three times.

### Western blotting

The expression levels of LAMP3, E-cadherin, and β-actin proteins were determined via western blotting. Cells were lysed with RIPA buffer (Beyotime Institute of Biotechnology) and protease inhibitor cocktail (Sigma-Aldrich). Then, proteins were added to each well of an SDS-PAGE setup. After protein concentration and separation, the proteins were carefully transferred to polyvinylidene fluoride (PVDF) membrane and treated with 5% non-fat dry milk in TBST buffer for blocking. After three washes, the membranes were incubated with primary antibodies at 4 °C overnight with shaking. The next day, the washed membranes were protected from light and exposed to HRP-conjugated secondary antibodies at room temperature for 1 h.

The protein levels were determined using a chemiluminescence substrate. In this section, the antibodies were: anti-LAMP3 antibody (Abcam, ab83659), cleaved caspase-3 (Asp175) antibody (Cell Signaling, 9661), mouse anti-β-actin (Abcam), anti-mouse HRP (Sigma), and anti-rabbit HRP (Sigma).

### Quantitative RT-PCR

To obtain total RNA, cells were lysed with TRIzol reagent (Invitrogen). The SuperScript III First-Strand Synthesis Kit (Invitrogen) was used to synthesize cDNA. Then quantitative RT-RCR was performed using a SYBR green master mix system (7900HT, Applied Biosystems). The 2^-ΔΔCt^ method was used to calculate the mRNA levels. The *GAPDH* gene was used as the internal control. The primer sequences were:

**Table:** 

Gene	Forward primer	Reverse primer
*LAMP3*	AGCAAGCACCTCACCAAACTT	TGTAGTCGCTGGGGTAGTTGT
*BIRC2*	AGCACGATCTTGTCAGATTGG	GGCGGGGAAAGTTGAATATGTA
*BIRC3*	TTTCCGTGGCTCTTATTCAAACT	GCACAGTGGTAGGAACTTCTCAT
*BNIP2*	TCCTAGTGATGGCTCTGTATTGT	ACTATTCTCTGACGGTGTGTCT
*BNIP3*	CAGGGCTCCTGGGTAGAACT	CTACTCCGTCCAGACTCATGC
*BNIP3L*	TTGGATGCACAACATGAATCAGG	TCTTCTGACTGAGAGCTATGGTC
*CASP3*	AGAGGGGATCGTTGTAGAAGTC	ACAGTCCAGTTCTGTACCACG
*CD27*	CAGAGAGGCACTACTGGGCT	CGGTATGCAAGGATCACACTG
*CD40LG*	ACATACAACCAAACTTCTCCCCG	GCAAAAAGTGCTGACCCAATCA
*BIRC6*	TAGTGTATGCCTCGTTTGTTGG	TTCTGTGTGTGCTCACCTTTC
*BRAF*	CCCCAAGTCACCACAAAAACC	CGGACTGTAACTCCACACCTT
*TP53*	CAGCACATGACGGAGGTTGT	TCATCCAAATACTCCACACGC
*CASP1*	TTTCCGCAAGGTTCGATTTTCA	GGCATCTGCGCTCTACCATC
*ABL1*	TGAAAAGCTCCGGGTCTTAGG	TTGACTGGCGTGATGTAGTTG
*AKT1*	AGCGACGTGGCTATTGTGAAG	GCCATCATTCTTGAGGAGGAAGT
*BAD*	CCCAGAGTTTGAGCCGAGTG	CCCATCCCTTCGTCGTCCT
*BAK1*	GTTTTCCGCAGCTACGTTTTT	GCAGAGGTAAGGTGACCATCTC
*BAX*	CCCGAGAGGTCTTTTTCCGAG	CCAGCCCATGATGGTTCTGAT
*BID*	ATGGACCGTAGCATCCCTCC	GTAGGTGCGTAGGTTCTGGT
*BIK*	GACCTGGACCCTATGGAGGAC	CCTCAGTCTGGTCGTAGATGA
*CASP10*	AGAAACCTGCTCTACGAACTGT	GGGAAGCGAGTCTTTCAGAAG
*CASP14*	CGCCTGGCCCTAATACTGTG	GGGTCTCTTTTCATGGTGCTTTC
*GAPDH*	GGAGCGAGATCCCTCCAAAAT	GGCTGTTGTCATACTTCTCATGG

### Apoptosis assay

An Annexin V-FITC/PI apoptosis Detection Kit (Abcam) was used to measure cell apoptosis. Cells were washed using PBS and then re-suspended in 200 μl binding buffer. After that, 5 μl Annexin V-FITC solution and 5 μl propidium iodide (PI) were incubated with the cells at 4 °C in the darkroom for 15 min. Samples were then analyzed via flow cytometry with Annexin V-(FL1-H) and PI-(FL2-H) on a BD FACSCalibur platform.

### Data acquisition

A tag cloud showing genes frequently mentioned to be associated with OS was acquired from the Osteosarcoma Database (http://osteosarcoma-db.uni-muenster.de/index.php). This database, which contains 911 protein-coding genes and 81 microRNAs associated with osteosarcoma (derived from 1331 abstracts), provides a structured view of the state of knowledge on osteosarcoma, relying on literature mining and manual annotation of PubMed abstracts [20]. The top genes related with OS can be searched and easily shown on the homepage of the website.

### Statistical analysis

All experiments were performed in at least three replicates. Data are presented as means ± SEM. Student’s t-test was analyzed to calculate the significance of means between two groups. Repeated analysis of variance (ANOVA) was performed to evaluate the difference in cell growth between the control and *LAMP3* or *TP53* siRNA groups. *p* < 0.05 was considered significant.

## Results

### *LAMP3* promotes OS cell viability

**Fig. 1:**
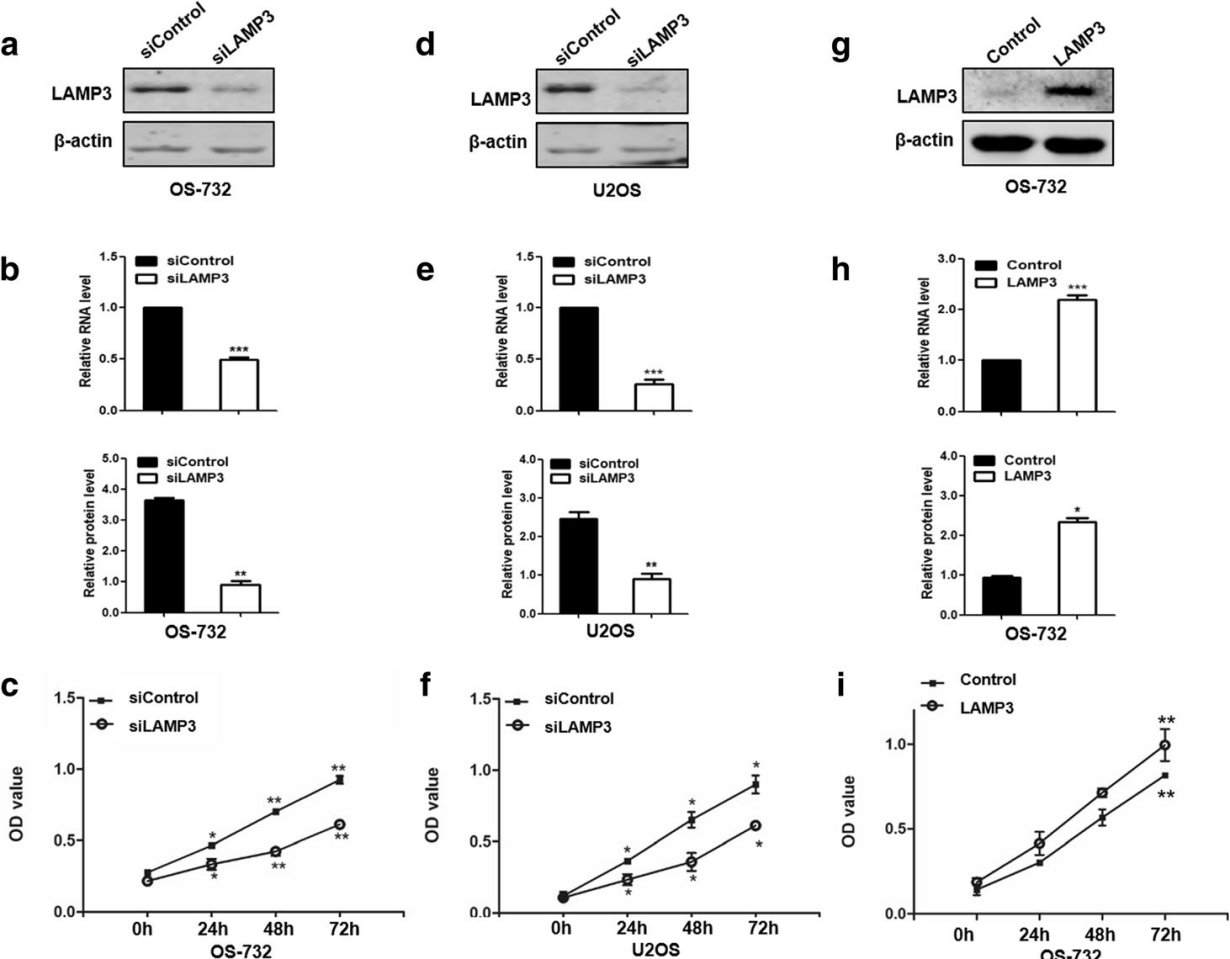
*LAMP3* regulated cell viability in osteosarcoma (OS) cells. **a**, **b**, **d** and **e** – Western blots showing the efficiency of *LAMP3* knockdown in OS-732 (**a**, **b**) or U2OS (**d**, **e**) cells at the mRNA (**a**, **d**) and protein (**b**, **e**) levels. β-actin served as the loading control. **g** and **h** – Western blots showing the efficiency of LAMP3 overexpression. **c**, **f** and **i** – MTT assay results showing that OS cell viability decreased after the inhibition of *LAMP3* (C) and increased following overexpression of *LAMP3* (**f**) compared with the control (**i**). *p < 0.05, **p < 0.01, ***p < 0.001, *n* = 3

To investigate the roles of *LAMP3* in OS cells, we transfected si*LAMP3* into OS cells and then examined cell viability. The mRNA and protein expression levels of *LAMP3* were significantly decreased after knockdown of *LAMP3* in OS-732 and U2OS cells (Fig. 1 and Additional file 1: Figure S1). Although cells in the si*LAMP3* group showed a minor growth increase, which may due to the gradual decrease in gene expression due to transient transfection over time, the cell viability was significantly inhibited. Overexpression of *LAMP3* led to evident increased cell proliferation, indicating that *LAMP3* is required for cell viability and promotes OS tumor cell growth.

### *LAMP3* inhibits OS cell apoptosis

**Fig. 2:**
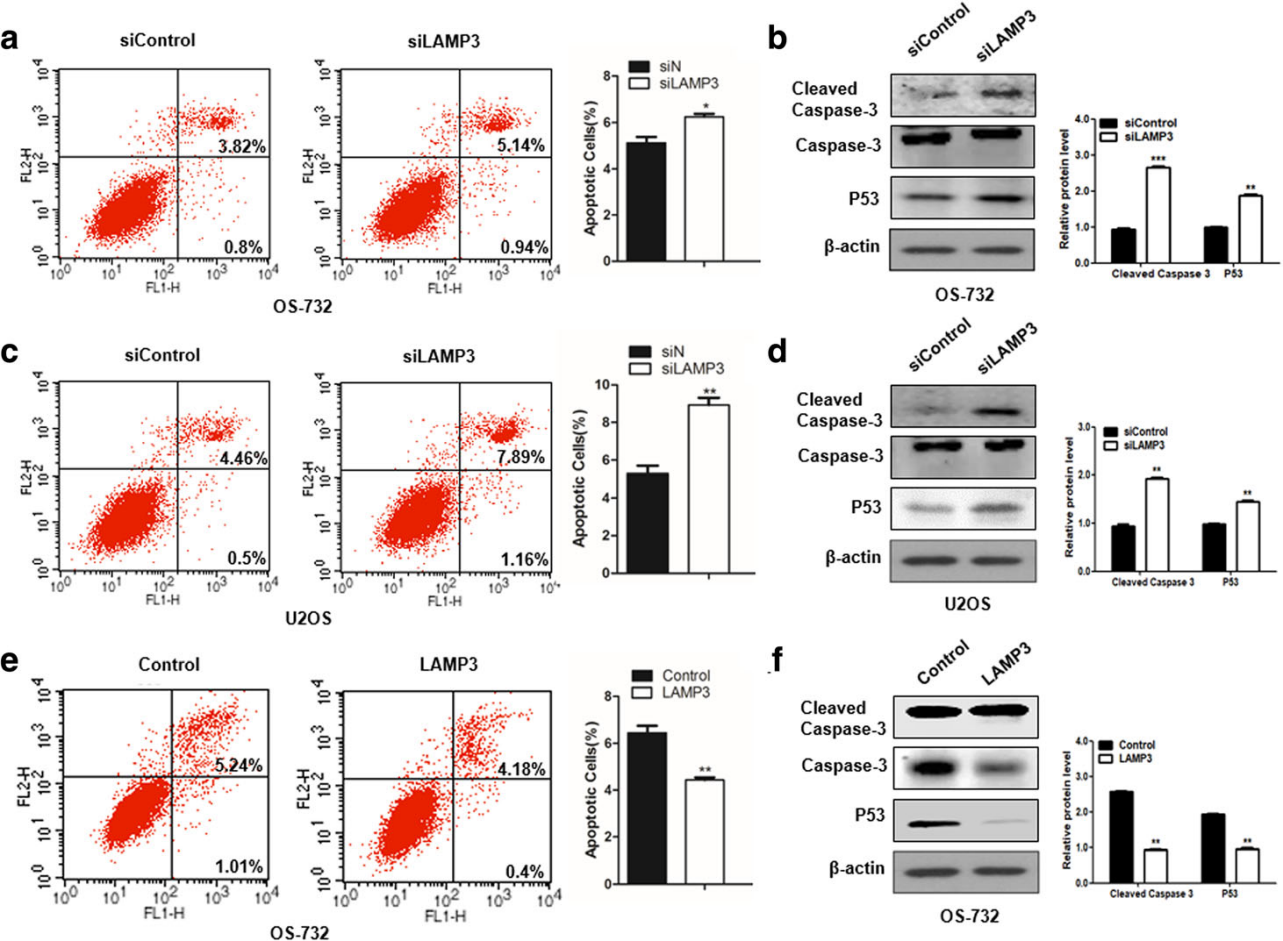
*LAMP3* regulated cell apoptosis in osteosarcoma (OS) cells. **a** and **c** – Apoptosis assay results showing that the percentage of apoptotic cells increased after inhibition of *LAMP3* compared with the control. E – The apoptotic rate significantly decreased when *LAMP3* was overexpressed compared with the control cells. **b** and **d** – Western blots showing the upregulated expression of cleaved-caspase-3 (**b**) and TP53 (**d**) after knockdown of *LAMP3* in either OS-732 or U2OS cells. **f** – Representative example of the downregulated expression of cleaved-caspase-3 and TP53 at the protein level with exogenous LAMP3 expression. *p < 0.05, **p < 0.01, ***p < 0.001, *n* = 3

To investigate how *LAMP3* affects cell viability, we assessed apoptosis after knockdown of *LAMP3*. As shown in Fig. 2, inhibition of *LAMP3* increased the expression level of cleaved caspase-3, a protein marker for the apoptosis in OS-732 and U2OS cells, suggesting that *LAMP3* might positively regulate OS cell viability by inhibiting cell apoptosis. Consistent with the western blot results for cleaved caspase-3, the percentage of late apoptotic cells increased from 3.82 to 5.14% in OS-732 cells and that of early apoptotic cells increased from 0.8 to 0.94%. Similar results were observed for si*LAMP3* U2OS cells. When we upregulated *LAMP3* expression in OS-732 cells, we found opposite effect. Overexpression of *LAMP3* inhibited the expression of cleaved caspase-3, and the apoptotic percentage decreased from 1.01 to 0.4% (early apoptosis) and 5.24 to 4.18% (late apoptosis) of cells. These results showed *LAMP3* could inhibit cell apoptosis.

### *TP53* is the top upregulated gene after knockdown of *LAMP3* in OS cells

**Fig. 3:**
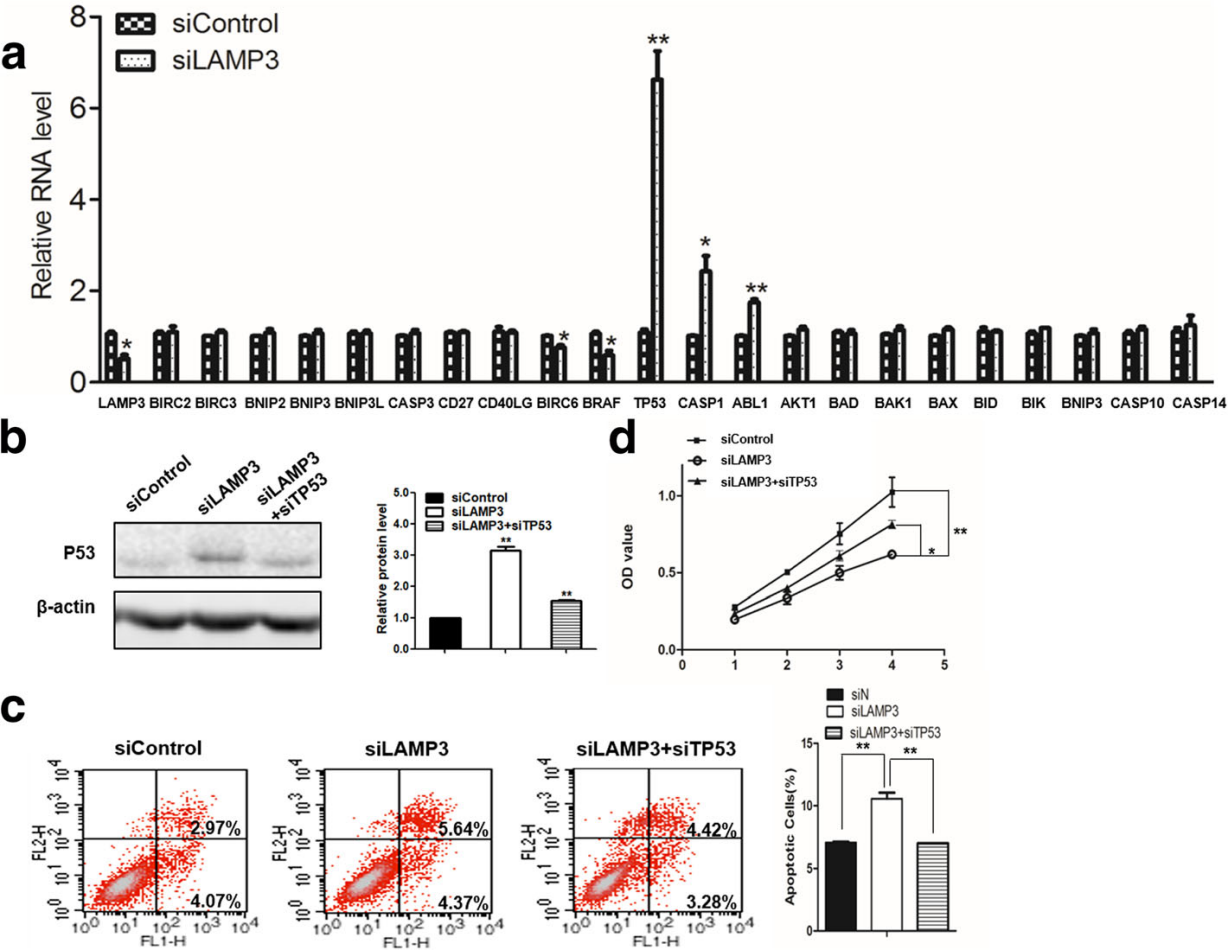
*TP53* was upregulated and partially reversed the effect of *LAMP3* on osteosarcoma (OS) cell viability and apoptosis. **a** – Quantitative RT-PCR results showing the mRNA levels of apoptotic genes after knockdown of *LAMP3*. **b** – Western blot confirmed the knockdown efficiency of TP53 with β-actin as the loading control. **c** – Apoptosis assay results showing that the percentage of apoptotic cells was decreased after inhibition of *TP53* in si*LAMP3* cells. **d** – MTT assay results showing that OS cell viability was increased after the inhibition of *TP53* in si*LAMP3* cells. *p < 0.05, **p < 0.01, ***p < 0.001, *n* = 3

To understand how *LAMP3* regulates OS cell apoptosis, we examined the apoptotic gene expression profiles by inhibiting *LAMP3* in OS cells. We observed that the mRNA levels of *TP53*, *CASP1* and *ABL1* significantly increased, while *BIRC6* and *BRAF* mRNA expressions significantly decreased (Fig. 3a). Those results suggest that these genes might play important roles in the *LAMP3*-mediated regulation of OS cell apoptosis. Notably, *TP53* was the top upregulated gene, indicating that *TP53* is the major response downstream gene of *LAMP3* in the regulation of OS cell apoptosis.

### Further knockdown of *TP53* partially reverses the effect of *LAMP3* on OS cell apoptosis and viability

To investigate the role of *TP53* in OS cell apoptosis promoted by inhibition of *LAMP3*, we further knocked down *TP53* in si*LAMP3* cells (Fig. 3b). We observed a similar increased percentage of late apoptotic cells, from 2.97 to 5.64% after knockdown of *LAMP3* in OS-732 cells, but this increase could be partially inhibited by further knockdown of *TP53*, whose ratio was 4.42% (Fig. 3c). Similar inhibition was also observed in early apoptotic cells (Fig. 3c). Consistently with the inhibition of cell apoptosis, the decreased cell viability in the si*LAMP3* group compared to the siControl group was also partially inhibited by the further knockdown of *TP53* (Fig. 3d). These results show that *TP53* is the key downstream gene of *LAMP3* in regulation of OS cell apoptosis and viability.

### *TP53* is top dysregulated gene in OS patients

**Fig. 4:**
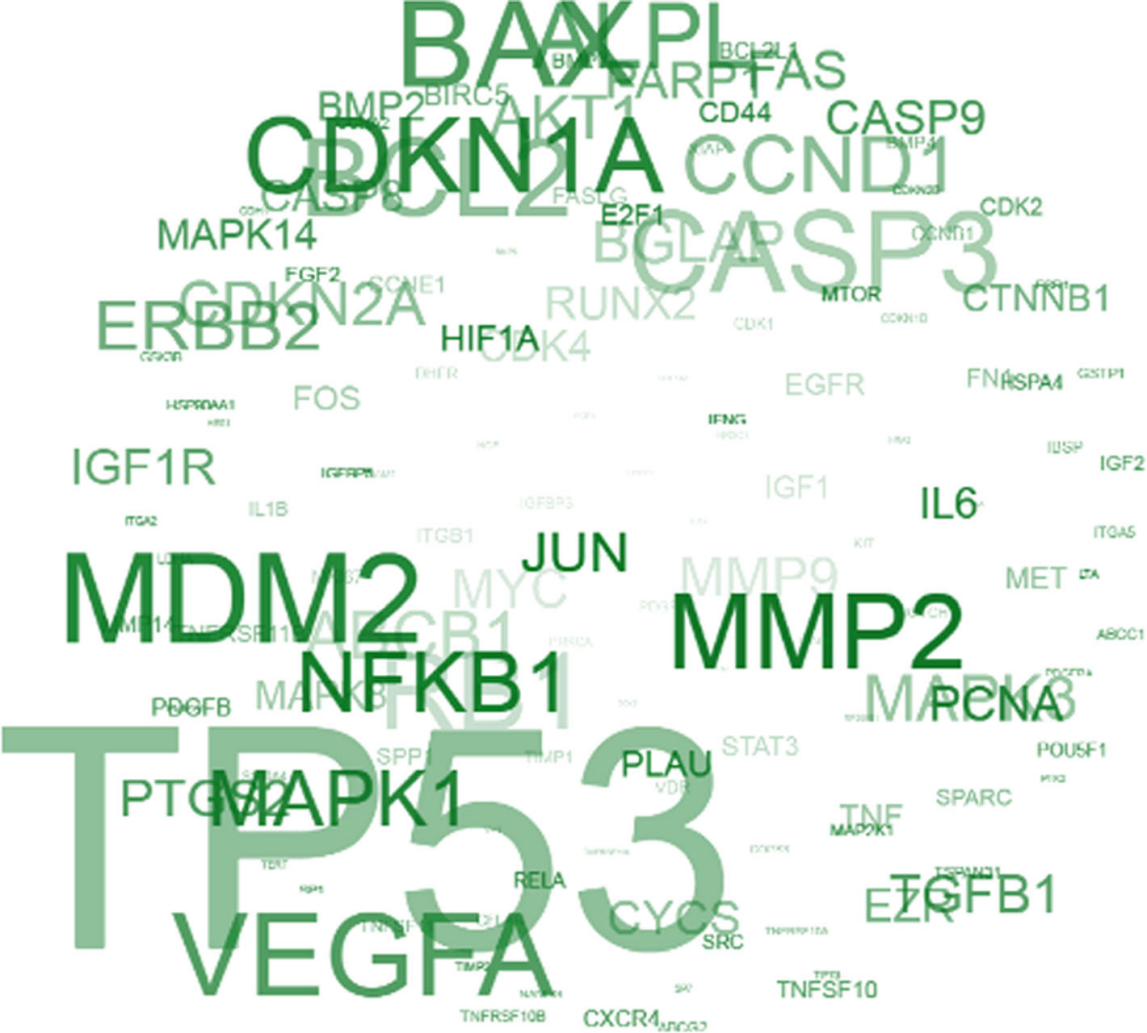
*TP53* is one of the top dysregulated genes in osteosarcoma (OS) patients according to the previous study (doi: 10.1093/database/bau042)S

To explore the clinical relevance of *TP53*, we examined the expression of *TP53* gene in OS patients in the online database. We found that *TP53* was a top dysregulated gene in OS patients (Fig. 4), representing genes frequently mentioned to be associated with OS. Those data suggest the strong clinical relevance of *LAMP3*’s key downstream gene, *TP53*, in OS patients. Therefore, activation of the TP53 pathway may be a promising way to treat OS in such patients.

## Discussion

Our previous study showed that *LAMP3* was significantly upregulated in osteosarcoma (OS) lung metastasis tissue compared to its expression in conventional OS tissue. Here, we showed that inhibition of *LAMP3* in OS cells reduced viability and increased apoptosis through the regulation of *TP53* expression. Therefore, activating TP53 pathway may be an alternative way to treat OS patients with higher levels of LAMP3.

It is reported that *TP53* mutations result in functional defects of tumor suppression, such as resistance to DNA damage and extensive proliferation [10], and enhanced proliferation, invasion and drug resistance in vitro [18]. Although the mechanisms of OS are complicated and include multiple genetic alterations, most OS progressions were caused by a preexisting genomic instability that is independent of *TP53* deficiency. Such instability could involve *TP53* and its regulated genes: *RB1*, *ATRX*, *DLG2* and the *PI3K/AKT/mTOR* pathway genes [11]. Importantly, polymorphisms of the *TP53* gene are associated with higher risk or survival for OS in the Chinese population [21].

In this study, we found that inhibition of *LAMP3* greatly increased the expression of *TP53* in OS cells. Silencing of *TP53* combined with si*LAMP3* did not fully restore the viability of OS cells, suggesting that other factors that could interfere with cell proliferation might occur following the double knockdown in OS cells. As a key tumor-associated gene, *TP53* might be a molecular target in clinical treatment of certain OS patients, e.g., those with *LAMP3* overexpression. However, Bisio demonstrated that LAMP3 itself is bound and transcriptionally activated by TP53 protein in the context of doxorubicin plus TNF-alpha induction [22], adding a layer of complexity to this system. Further investigation is needed.

We also detected other upregulated genes in si*LAMP3* OS cells, including *CASP1* and *ABL1*. Caspase-1 is an inflammatory caspase. Once activated, its downstream executioner caspases can be induced, which is pivotal to induce apoptosis [23]. *ABL1* is a proto-oncogene encoding tyrosine kinase. Its expression level is associated with the formation of hematopoietic malignancy and the regulation of apoptosis in T-lymphocytes [24]. Extensive studies have demonstrated that *ABL1* can work as a co-activator and positive cofactor of *TP53* in activating genes involved in cell cycle arrest and apoptosis in cases of DNA damage [25–27].

We also found that *BIRC6* and *BRAF* were downregulated after knockdown of *LAMP3*. *BIRC6* is a member of the inhibition of apoptotic proteins (IAPs) family, and it regulates the degradation of caspase-9. It negatively regulates apoptosis by facilitating the degradation of *TP53* [28]. *BRAF* usually serves as a biomarker for many tumors, including colorectal cancer, urachal carcinoma and melanoma cells [29]. This is consistent with its expression pattern in the inhibition of *LAMP3* in OS cells. Therefore, these less changed genes, might also contribute to the regulation of *LAMP3* in OS cell apoptosis and viability. Further studies are needed to classify it.

Future research should also be considered to reveal the details of: *LAMP3* regulation of *TP53* expression in OS cells; the in vivo phenotypes; the interplay between those downstream genes and *LAMP3*; and the other mechanisms of *LAMP3* in the regulation of OS cell viability.

## Conclusions

Our study showed that *LAMP3* promotes OS cell viability and inhibits apoptosis. *TP53* was found to be the top upregulated gene upon *LAMP3* knockdown. Further knockdown of *TP53* partially reverse the effect of *LAMP3* on OS cell viability and apoptosis. Human clinical data showed *TP53* was the top dysregulated gene in OS patients. Thus, activating *TP53* may be a good clinical approach to treat OS patients with higher expression of *LAMP3*.

## Additional file

Additional file 1: **Figure S1.** A – The full image of the western blot of LAMP3 detection with anti-*LAMP3* from Abcam (#ab83659) in osteosarcoma (OS) cells. The rectangular box outlines the LAMP3 protein bands shown in Fig. 1a and d. B – Western blot analysis showed that LAMP3 protein expression was sharply inhibited by tunicamycin treatment in U2OS cells. (TIF 547 kb)

## Abbreviations

IAPs: Inhibition of apoptotic proteins
LAMP3: Lysosome-associated membrane glycoprotein 3
OS: Osteosarcoma
PVDF: Polyvinylidene fluoride
