# Supplementary figures and images for: LAMP3 plays an oncogenic role in osteosarcoma cells partially by inhibiting TP53
Source: Cell Mol Biol Lett. 2018 Jul 11;23:33. doi: 10.1186/s11658-018-0099-8 (PMC6042264; doi:10.1186/s11658-018-0099-8)

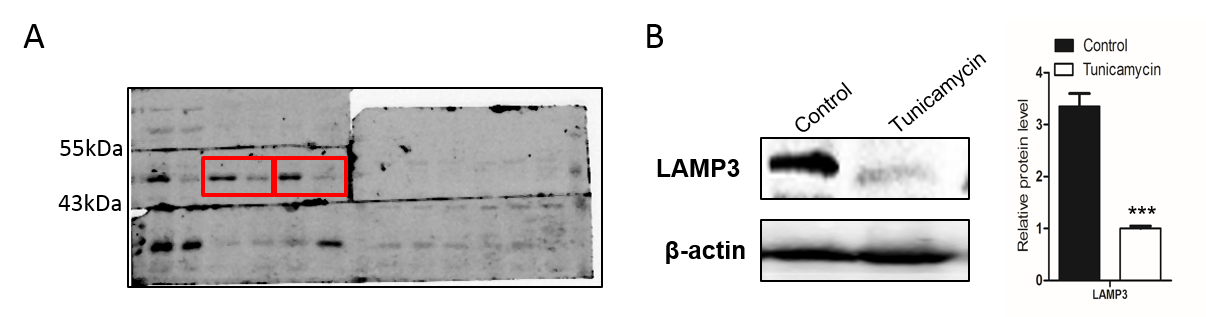

Supplement: Supplementary file 1 — Figure S1. A – The full image of the western blot of LAMP3 detection with anti-LAMP3 from Abcam (#ab83659) in osteosarcoma (OS) cells. The rectangular box outlines the LAMP3 protein bands shown in Fig. 1a and d. B – Western blot analysis showed that LAMP3 protein expression was sharply inhibited by tunicamycin treatment in U2OS cells. (TIF 547 kb) [file 11658_2018_99_MOESM1_ESM.tif]
